# Strict Versus Moderate Versus Liberal Dietary Sodium Restriction in Chronic Heart Failure: A Systematic Review and Meta-Analysis of Randomized Controlled Trials

**DOI:** 10.3390/jcm15218710

**Published:** 2026-11-08

**Authors:** Rabia Safiya, Alaa Safia, Ashraf Abu Shkara

**Affiliations:** 1 Department of Internal Medicine, Ziv Medical Center, Safed 1310001, Israel; 2 Department of Otolaryngology, Ziv Medical Center, Safed 1310001, Israel; 3 The Azrieli Faculty of Medicine, Bar-Ilan University, Safed 1311502, Israel

**Keywords:** heart failure, dietary sodium restriction, sodium intake, heart failure hospitalization, quality of life, NYHA functional class

## Abstract

**Background:** Clinical trials on dietary sodium restrictions among heart failure (HF) patients have provided inconsistent findings with limited evidence of major benefits and risks of sodium intake restrictions. This systematic review and meta-analysis compared the impact of strict versus moderate versus liberal sodium intake on all-cause mortality, HF hospitalization and composite outcomes in HF patients. **Methods:** A systematic search for randomized controlled trials (RCTs) was conducted across Embase, MEDLINE, and Cochrane Library databases in May 2026 and updated in June 2026. Risk of bias assessment was conducted using the Cochrane Risk of Bias (RoB 2) tool and certainty of evidence assessed using the GRADE framework. Meta-analysis using random-effects model was conducted to pool effects across included studies. **Results:** A total of 10 RCTs met the inclusion criteria. Strict and moderate sodium restriction strategies did not significantly reduce all-cause mortality (OR = 0.53; 95% CI: 0.10–2.69; *I*^2^ = 18.3%), HF hospitalization (RR = 0.84; 95% CI: 0.62–1.15; *I*^2^ = 0%) or composite outcome of death/HF hospitalization (OR = 0.92; 95% CI: 0.67–1.26; *I*^2^ = 0%) compared to liberal sodium restriction. However, strict to moderate sodium intake was associated with improved quality of life (QoL) (SMD = 0.33; 95% CI: 0.15–0.51; *I*^2^ = 0%), New York Heart Association (NYHA) functional class improvement (OR = 1.51; 95% CI: 1.18–1.94; *I*^2^ = 0%) and reduced BNP/NT-proBNP (ratio = 0.62; 95% CI: 0.42–0.90; *I*^2^ = 57.2%) compared to liberal intakes. **Conclusions:** Compared with higher sodium intake, lower sodium targets did not demonstrate significant reductions in all-cause mortality or HF hospitalization in the available RCTs. Lower sodium intake was associated with improvements in quality of life, NYHA functional class, and BNP/NT-proBNP; however, the certainty and generalizability of these findings remain limited. Current evidence is therefore insufficient to establish an optimal dietary sodium target for patients with heart failure, and adequately powered randomized trials comparing clearly defined sodium targets are warranted.

## 1. Introduction

Heart failure (HF) is increasingly becoming a global health burden. The disease arises due to structural or functional impairments leading to the heart’s inability to effectively pump blood needed to meet metabolic demand [1]. Ischemic heart disease is the leading cause of HF, with other causes including hypertension, myocarditis and vascular diseases [2]. Acute myocardial infarction or hypertensive emergency can occur in stable patients who present with stable chronic HF symptoms, causing their condition to decompensate rapidly and trigger worsened HF symptoms like acute pulmonary edema [3]. The classification of HF is crucial in determining the management approach. The New York Heart Association (NYHA) stratifies HF patients based on functional capacity and severity of HF symptoms, while the American Heart Association (AMA) bases its classification on left ventricular ejection fractions (LVEF) [4]. Based on NYHA guidelines, HF patients are classified as having low or reduced ejection fraction (HFrEF: LEVF ≤ 40%), preserved ejection fraction (HFpEF: LEVF ≥ 50%) or mid-range ejection fraction (HFmrEF: LEVF 40–50%). Epidemiological trends indicate reduced prevalence of HFrEF due to better management but rising prevalence of HFpEF [5]. This trend has been linked to increasing cardiometabolic risk factors, including obesity, diabetes and hypertension. Despite differences in ejection fractions, both HFrEF and HFpEF demonstrate comparable hospitalization and mortality rates [6].

The management of HF is multifaceted, combining pharmacological approaches such as medications and therapy, and non-pharmacological interventions such as education and dietary restriction [7]. Sodium dietary restriction is a widely recommended intervention for chronic HF management. The benefits of salt restrictions in HF patients are well established in clinical practice. Several observational studies have suggested that maintaining sodium intake between 1500 mg and 2300 mg a day in chronic HF patients helps avoid congestions and reduces cardiovascular events by 25% in the long-term [8]. Sodium reduction has been shown to mitigate fluid retention, reduce preload and help achieve better blood pressure control [9,10]. Despite benefits, the impact of different sodium intake levels on clinical outcomes in HF patients is inconsistent. Balancing sodium intake levels and long-term metabolic health is a long-debated issue.

Early studies have reported that restricting sodium intake leads to symptomatic relief for HF patients [8]. However, more recent studies have shown that excessive sodium intake is strongly linked with high blood pressure, hypertension and impaired renal function in HF patients [11]. Further, overly strict sodium restrictions can trigger dangerous hormonal changes, worsening outcomes in HF patients [12]. The evidence supports moderate sodium intake ≤ 2300 mg/day to improve metabolic health and reduce cardiovascular risks. However, the findings also highlight variability in patient handling of sodium intake and resulting impact on metabolism and hemodynamic fluctuations, depending on sodium sensitivity.

Clinical trials on dietary sodium restrictions among HF patients have provided inconsistent findings on primary outcomes, including all-cause mortality and HF hospitalization or mortality rates and secondary outcomes such as BP, quality of life (QoL) and renal function [13,14]. Systematic reviews and meta-analyses have reported major benefits and risks of sodium intake restrictions [15,16,17].

Previous systematic reviews and meta-analyses have evaluated dietary sodium restriction in patients with heart failure. However, uncertainty remains regarding the comparative effects of different sodium-intake targets and whether the effects vary according to heart failure phenotype and clinical setting. Most studies have treated dietary sodium intake as a single, homogeneous intervention, pooling trials with widely different prescribed targets and thereby obscuring clinically relevant intake targets. Subgroup evidence by phenotype and setting is limited, while patient-centered outcomes such as NYHA functional class and quality of life are rarely synthesized or inconsistently reported. The present systematic review was therefore designed to extend the existing evidence by restricting the quantitative synthesis to randomized controlled trials and by evaluating clinically distinct sodium-intake targets rather than considering sodium restriction as a single intervention. In addition to major clinical outcomes, including all-cause mortality and heart failure hospitalization, we evaluated patient-centered and surrogate outcomes, including quality of life, NYHA functional class, and B-type natriuretic peptide and N-terminal pro-B-type natriuretic peptide (BNP/NT-proBNP). We further examined the robustness and sufficiency of the evidence using prespecified subgroup analyses, sensitivity analyses excluding acute/inpatient heart failure populations, meta-regression, Grading of Recommendations Assessment, Development and Evaluation (GRADE) assessment, and trial sequential analysis. Accordingly, the objective of this systematic review and meta-analysis was to compare the effects of different dietary sodium restriction strategies on clinical and symptom-related outcomes among patients with heart failure.

## 2. Materials and Methods

### 2.1. Protocol Registration

The study protocol was developed following the Preferred Reporting Items for Systematic Reviews and Meta-Analyses (PRISMA) guidelines [16]. A comprehensive literature search, article selection, and data extraction strategy were formulated and executed to synthesize the available evidence and perform a comparative analysis of sodium intake and chronic heart failure. The protocol was registered with the PROSPERO CRD registration number CRD420261426861. The reporting of this systematic review followed the PRISMA 2020 checklist presented in Table 1.

### 2.2. Databases and Search Strategy

A comprehensive literature search was conducted in three databases: Embase, MEDLINE and the Cochrane Register of Controlled Trials (CENTRAL) in May 2026 and updated in June 2026. We combined controlled vocabulary and free-text keywords relating to dietary sodium restrictions, sodium intake levels and chronic heart failure, which were applied to the databases to aid in the search. The search query was (“Heart failure” OR “cardiac failure”) AND (“sodium, dietary” OR “dietary sodium restriction” OR “sodium”) AND (“intake” OR “diet” OR “restriction” OR “low sodium” OR “moderate sodium” OR “liberal sodium”). Study design filters were also added to the query (“Randomized controlled trials” OR “RCTs”). The search query was adapted to the syntax and controlled vocabulary of each database. A randomized controlled trial filter was applied and the search was limited to studies published in English with no restriction on the year of publication. In addition, we searched ClinicalTrials.gov and the WHO International Clinical Trials Registry Platform (ICTRP) for ongoing or unpublished randomized controlled trials (RCTs) involving dietary sodium restrictions in heart failure. We manually screened the reference lists of included studies and relevant reviews to identify additional eligible studies.

### 2.3. Screening and Selection Criteria

**Inclusion and exclusion criteria**

The literature search targeted RCTs that involved adult patients with chronic heart failure, predominantly in ambulatory or stable outpatient settings. Trials enrolling patients during acute decompensated heart failure (ADHF) were considered if outcomes were assessed after stabilization.

**Inclusion criteria included:** RCTs that reported sodium intake levels and clinical outcomes, particularly all-cause mortality, HF hospitalization rates, mortality rates, blood pressure, quality of life and renal function were included. Most included trials compared low versus usual/higher sodium diets using thresholds around 2000 mg/day to 5000 mg/day. This analysis evaluated three prespecified sodium intake categories: strict (<1500 mg/day), moderate (1500–2300 mg/day), and liberal (>2300 mg/day). We mapped each trial arm to one of these sodium intake categories primarily on the basis of the prescribed targets or achieved mean/median intake where prescribed targets were not reported. Where both prescribed target and achieved intake were reported, we used the prescribed target as the primary basis for classification and noted instances in which prescribed intake appeared to fall into a different sodium category.

**Exclusion criteria were defined as follows:** Non-randomized trials were excluded from this review. Studies that did not report all-cause mortality, HF hospitalization rate, mortality rate, BP, QoL or renal function, were also excluded. Non-English studies, systematic reviews and conference abstracts were excluded.

### 2.4. To Data Extraction and Risk Assessment

Two independent reviewers screened the titles, abstracts and full texts of selected studies against the eligibility criteria. Studies that met the inclusion criteria were selected for inclusion in the review.

The reviewers independently extracted data from the included studies using a standardized form. The extracted data included study and population characteristics, dietary sodium intake levels and outcomes such as all-cause mortality, HF hospitalization rate, mortality rate, blood pressure, QoL, renal function and adverse events. Discrepancies were solved through consultation or consensus.

**Quality assessment**

The methodological quality and risk of bias of the RCTs were assessed at outcome level using the Cochrane Risk of Bias 2 (RoB 2) tool [18]. The tool assess risk of bias across domains such as randomisation process, deviation from intended interventions, missing outcome data, measurement of outcomes and selection of results reported. Risk of bias was categorized as low, some concerns or high depending on the scores. The quality of evidence was assessed using the Grading of Recommendations, Assessment, Development and Evaluation (GRADE) criteria [19]. The quality was assessed across domains such as risk of bias, inconsistency, imprecision and publication bias.

**Statistical analysis**

Meta-analysis was conducted using random-effects models to calculate the pooled effect sizes on the association between sodium intake and metabolic endpoints and cardiovascular risk outcomes. All statistical analyses were performed using R Software Version 4.6.1. For dichotomous outcomes, the effect measure used for each analysis was prespecified according to the available event data and the statistical model applied. Odds ratios (ORs) with 95% confidence intervals (CIs) were used for all-cause mortality, the composite outcome of death/HF hospitalization, and improvement in NYHA functional class, whereas risk ratios (RRs) with 95% CIs were used for HF hospitalization. For quality of life, standardized mean differences (SMDs) with 95% CIs were calculated because different measurement scales were used across studies. BNP/NT-proBNP was synthesized as a ratio measure. The same effect measure was used consistently for each outcome throughout the analyses, figures, and reporting.

For continuous outcomes like quality of life, standardized mean differences (SMDs) were calculated with 95% confidence intervals. Heterogeneity was examined using the *I*^2^ statistic and Chi-Square [20]. Random-effects meta-regression analyses were performed using the restricted maximum likelihood (REML) method. Prespecified covariates included sodium intake targets, HF phenotypes and clinical settings. Subgroup analysis was conducted based on HF classification (HFrEF-only versus mixed HFrEF/HFpEF populations) and sodium intake levels (<1500 mg/day vs. 1500–2300 mg/day vs. >2300 mg/day). To assess the robustness of the meta-analysis findings and quantify the risk of random errors due to sparse data, trial sequential analysis (TSA) was performed for the primary outcomes, all-cause mortality and HF hospitalization. TSA modeling used a two-sided type I error (α) of 5%, type II error (β) of 20% (80% power), and an anticipated relative risk reduction (RRR) of 20% for each endpoint. Endpoint-specific pooled control event rates were used: 4% for all-cause mortality and 12% for HF hospitalization. Because these baseline risks differ materially, the required information size (RIS) was recalculated separately for each endpoint using the conventional two-group binary-outcome information-size formula under equal allocation: Approximately 17,022 participants for all-cause mortality and 5250 participants for HF hospitalization. With 1074 participants currently available, the corresponding additional sample sizes are approximately 15,948 and 4176 participants, respectively. These RIS values are planning estimates based on the stated α, power, control event rate and 20% RRR assumptions; they are not a single shared RIS across endpoints. A sensitivity analysis was also conducted excluding inpatient/acute HF studies. eave-one-out analysis was conducted to assess the influence of RCTs using the DerSimonian-Laird method, sequentially excluding each included study. A funnel plot and Egger’s test were used to explore potential publication bias and small-study effects.

Because follow-up duration varied substantially across trials (15 days to 12 months), outcomes were pooled at available endpoint effect estimates when studies were sufficiently comparable in terms of all-cause mortality, HF hospitalization, composite outcomes, QoL and NYHA class. This approach was necessary because time-to-event, person-time or common time-point data and sufficiently standardized time-specific outcome reporting were unavailable. Given the clinical relevance of unequal observation periods, particularly to mortality, HF hospitalization and composite outcome, these estimates represent effects observed across the range of follow-up durations studied rather than effects at a single point.

## 3. Results

### 3.1. Characteristics of Included Studies

**Figure 1:**
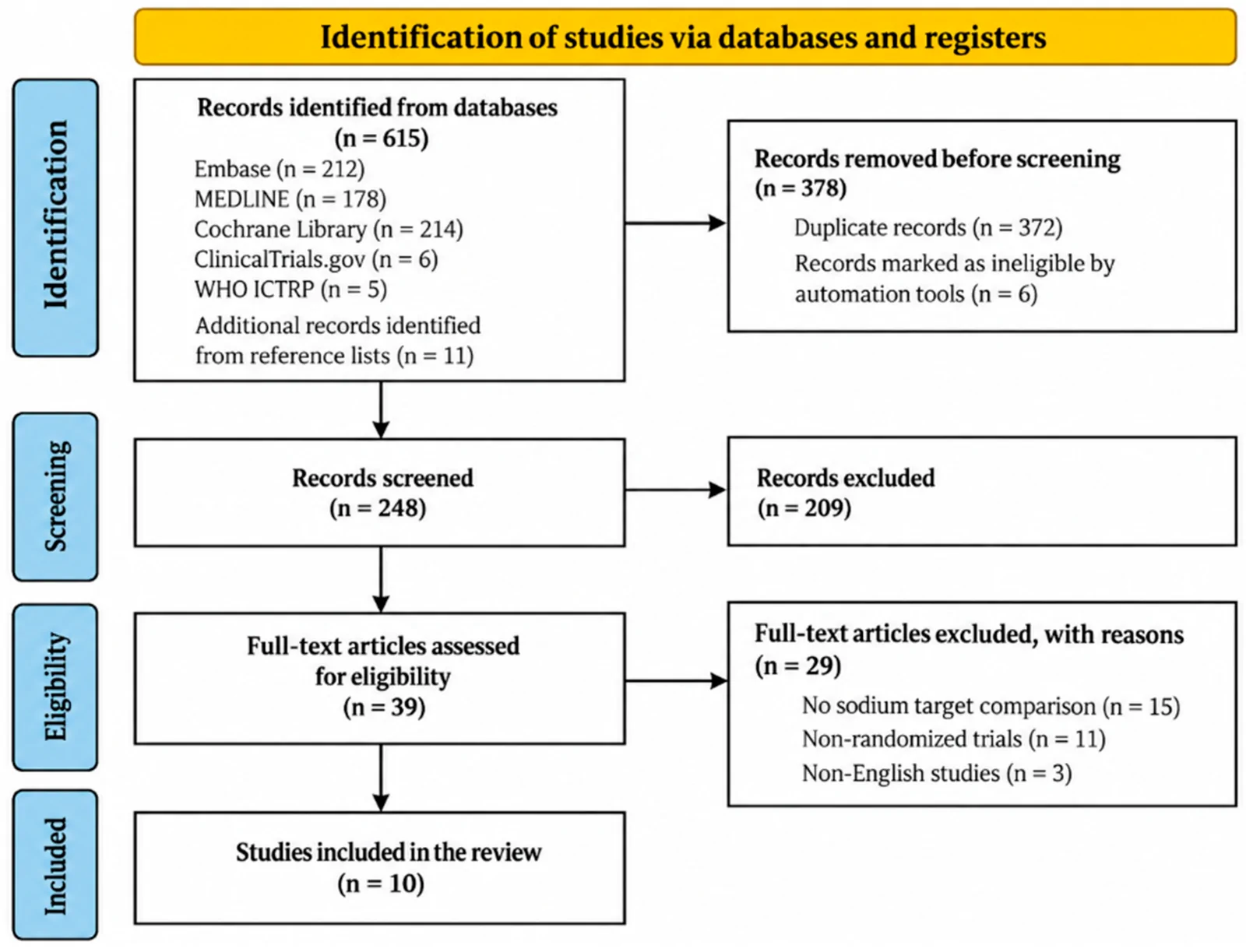
The PRISMA (Preferred Reporting Items for Systematic Reviews and Meta-Analyses) guidelines illustrating the study selection process, which was aligned with the methodological principles recommended in the Cochrane Handbook for Systematic Reviews.

The initial search of databases and reference lists of included studies yielded 626 records, of which 378 duplicates were removed. The screening of titles and abstracts of remaining studies led to the exclusion of 209 studies. Full texts screening of the remaining 39 studies against the eligibility criteria resulted in the exclusion of 29 studies. Only 10 studies met the inclusion criteria. Sensitivity analyses were conducted to evaluate the robustness of the pooled estimates by excluding studies involving acute decompensated HF, and smaller studies ≤ 30 patients. Leave-one-out analyses were also performed excluding each study, including the largest studies (SODIUM-HF). Meta-regression analysis was performed to examine potential sources of heterogeneity. A Trial Sequential Analysis (TSA) was done to assess whether cumulative evidence for mortality and HF hospitalization was sufficient to make conclusive inferences. Information size was calculated using α = 0.05, β = 0.20 and relative risk reduction of 20% (Figure 1).

**Study Characteristics**

Across the included trials, prescribed sodium targets varied substantially. For the purpose of subgroup analyses, intervention targets were categorized as strict (<1500 mg/day), moderate (1500–2300 mg/day), or liberal/usual (>2300 mg/day). Because individual trials compared different pairs of sodium targets, the primary meta-analyses were interpreted as comparisons of lower versus higher prescribed sodium intake, whereas target-specific analyses were examined separately. The setting was predominantly outpatients with one RCT involving inpatients and another involving post-discharge patients. The sample size ranged from as small as 12 to large trials of 800 patients. The average age ranged between 55 and 77 years, and the population was predominantly male. Strict sodium restriction ranged from 800–1500 mg/day, moderate restriction ranged from 1800–2300 mg/day, while usual care restrictions ranged from 2300–5000 mg/day. Follow-up duration ranged between a short-term of 15 days to a long-term of 12 months. (Table 2).

### 3.2. Risk of Bias

**Figure 2:**
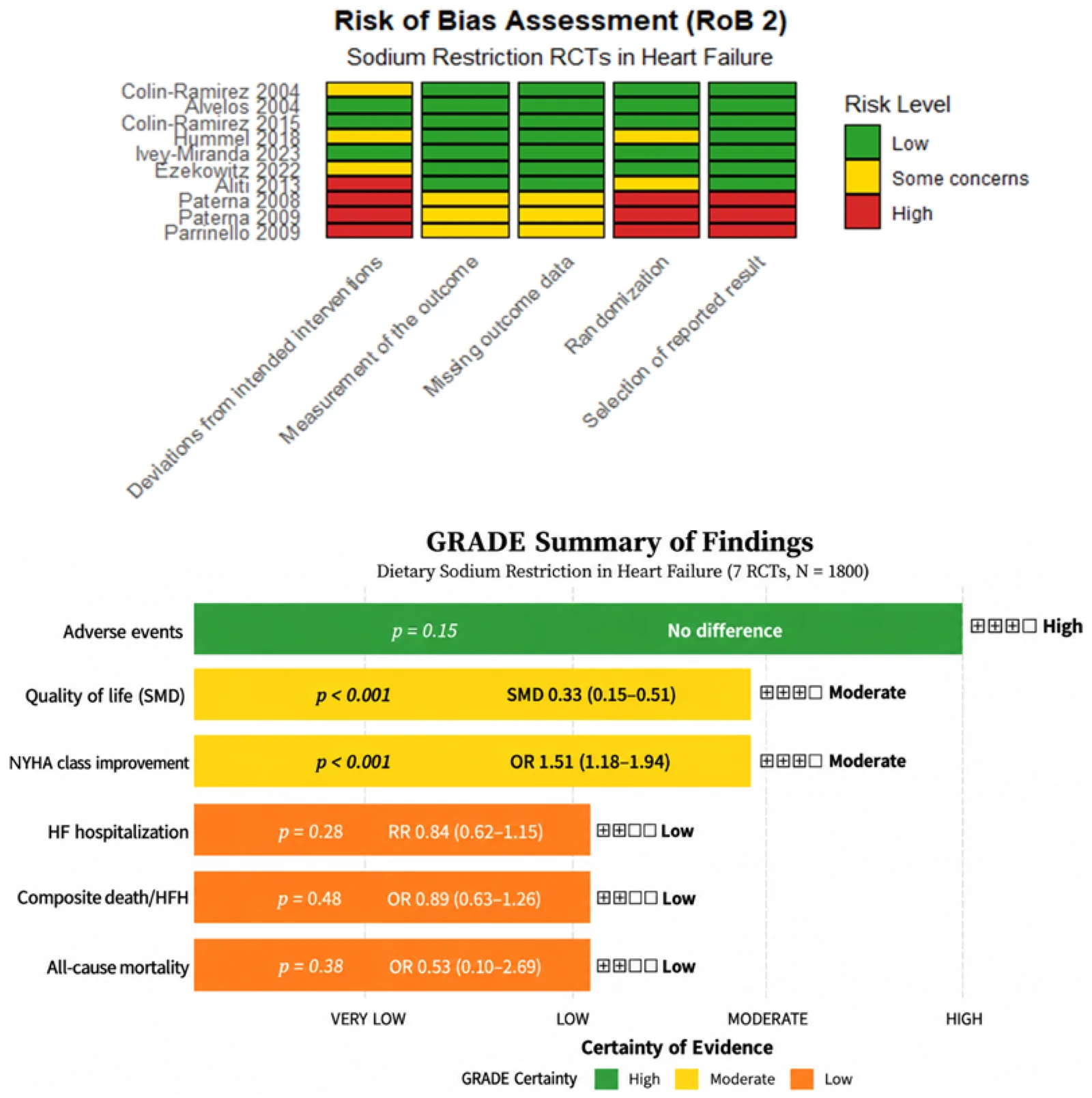
Risk of Bias using ROB2 and GRADES. Risk of Bias Assessment (RoB 2) for the included randomized controlled trials: Colin-Ramirez et al. (2004) [21], Alvelos et al. (2004) [22], Colin-Ramirez et al. (2015) [23], Hummel et al. (2018) [24], Ivey-Miranda et al. (2023) [25], Ezekowitz et al. (2022) [26], Aliti et al. (2013) [27], Paterna et al. (2008) [28], Paterna et al. (2009) [29], and Parrinello et al. (2009) [30]. Green indicates low risk, yellow indicates some concerns, and red indicates high risk.

The risk of bias analysis was performed on the 10 RCTs with judgements made at the outcome-level for the five domains including the randomization process, deviations from intended interventions, missing outcome data, measurement of the outcome and selection of the reported results (Figure 2). The results showed a low to high risk of bias across the five RoB 2 domains. Seven of the studies demonstrated a low risk across all domains, while three studies were judged to have some concerns or a high risk of bias mainly due to issues in deviations from intended interventions, randomisation, selection of reported results, measurement of the outcome and missing outcome data. One study was judged to be at high risk for deviations from intended deviations. Overall, most studies were judged as having a low risk of bias although three earlier studies raised some concerns to high risk of bias regarding methodological quality.

The GRADE assessment showed moderate certainty of evidence that dietary sodium restriction improved QoL and NYHA functional class. However, certainty was low that sodium restriction impacts all-cause mortality, HF hospitalization and composite death or HFH, mainly due to imprecision, risk of bias and inconsistency for some outcomes. Most trials were judged to be at low risk of bias although several had some concerns or a high risk of bias in key domains including deviations from intended deviations, randomisation, and selection of reported results. Table 3 provides the GRADE summary of findings showing the effect estimates, anticipated absolute effects per 1000 participants, and certainty of evidence for the outcomes. Overall, the GRADE assessment indicated low to moderate certainty of evidence, suggesting varying clinical benefits of dietary sodium restriction. The findings indicate that while sodium dietary restrictions may offer improvements in QoL and NYHA functional class, evidence on hard clinical outcomes remains inconclusive. (Figure 2).

### 3.3. Meta-Analysis

**Meta-Analysis of RCTs**

All 10 RCTs were included in the meta-analyses of metabolic health and cardiovascular risks with sodium dietary restrictions in HF patients. The RCTs used lower sodium intakes (800–2000 mg/day) in the intervention groups and high intakes (2200–3000 mg/day) in the control groups. In all arms, for which both prescribed targets and achieved intake were reported, the achieved intake was consistent with the category assigned on the basis of the prescribed target.

**All-cause mortality**

**Figure 3:**
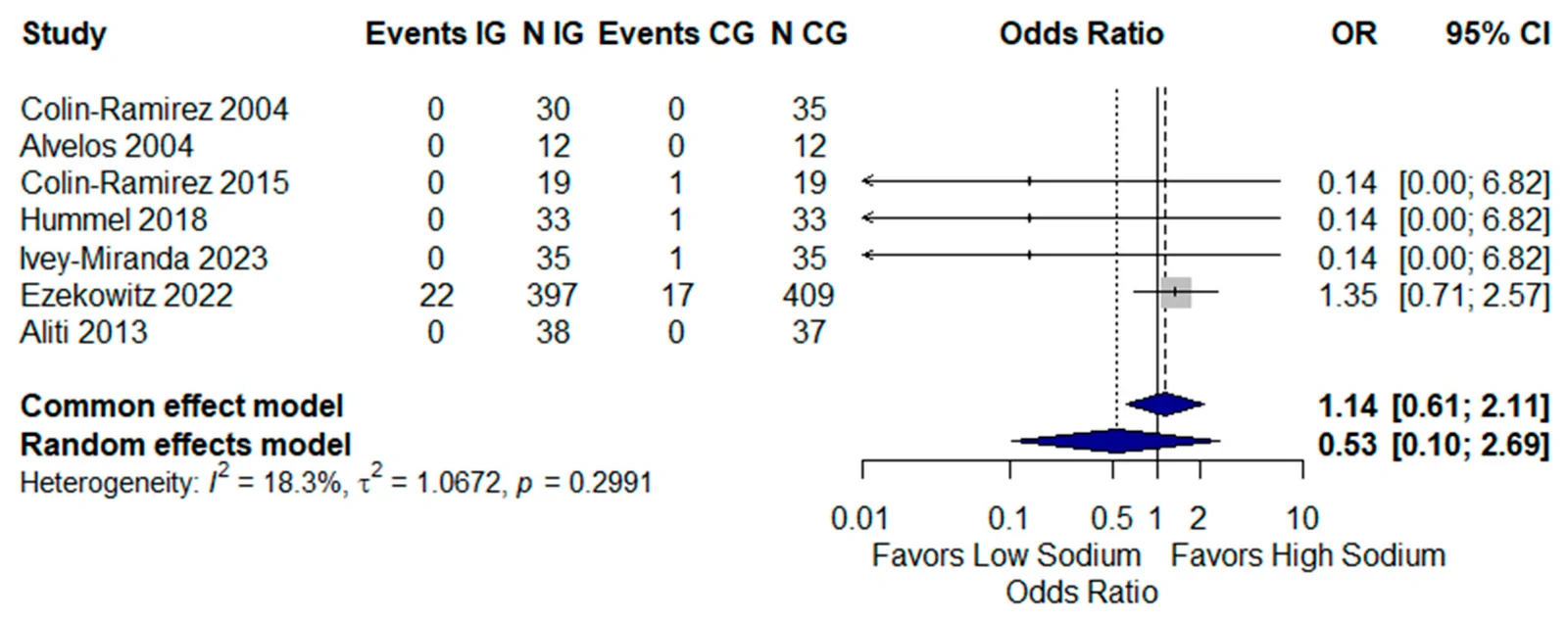
Forest plot of all-cause mortality. Forest plot of all-cause mortality. The trials shown include Colin-Ramirez et al. [21], Colin-Ramirez et al. [23], Ezekowitz et al. [26], Ivey-Miranda et al. [25], Hummel et al. [24], Alvelos et al. [22] and Aliti et al. [27].

Seven RCTs were included in a pooled analysis of all-cause mortality using a random-effects model comparing lower versus high dietary sodium intake. The pooled effects showed an odds ratio of OR = 0.53 (95% CI: 0.10–2.69) suggesting lower odds of all-cause mortality with strict and moderate sodium intake compared to liberal intake, although the results were not statistically significant. The confidence interval included 1, and no statistically significant benefit could be demonstrated. Heterogeneity was low, *I*^2^ = 18.3% (*p* = 0.30), suggesting variability between trials. These findings show that strict and moderate sodium restriction provides no clear benefit over liberal intakes in HF patients. Several studies reported few or no mortality events which may have influenced the effect estimates, hence pooled estimates should be interpreted cautiously (Figure 3).

**HF Hospitalization**

**Figure 4:**
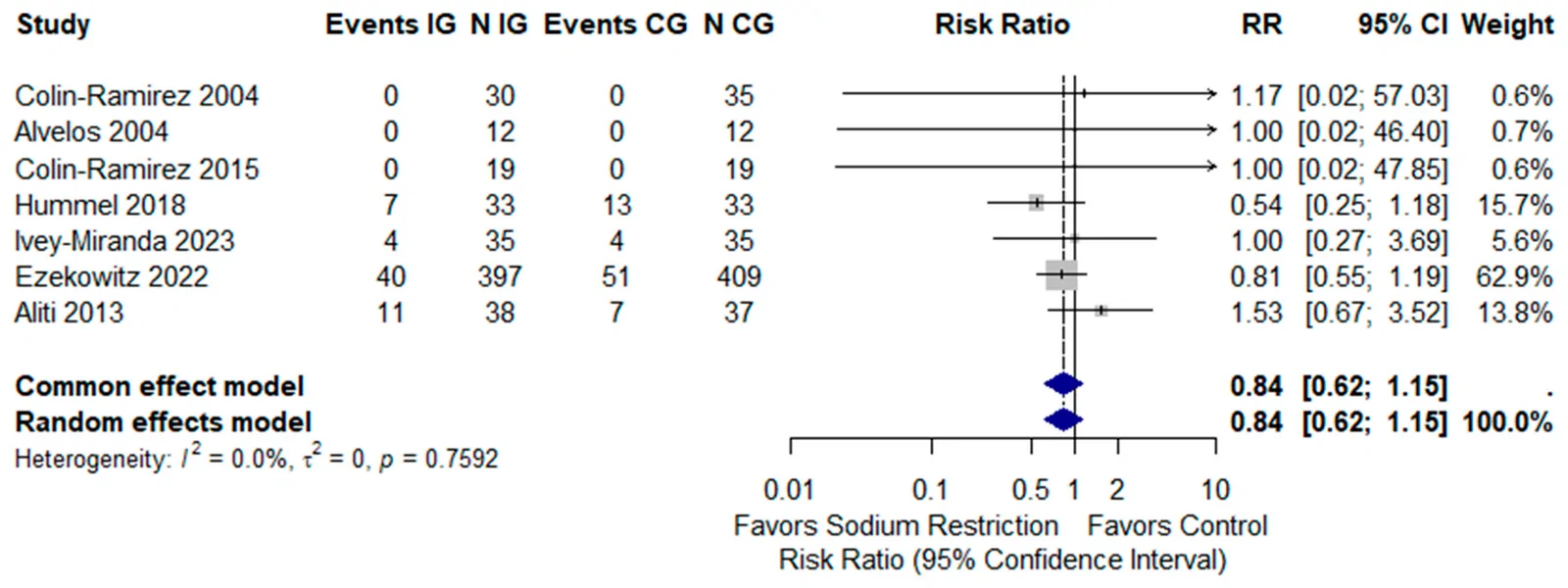
Forest plot of heart failure hospitalization. The trials shown include Colin-Ramirez et al. [21], Colin-Ramirez et al. [23], Ezekowitz et al. [26], Ivey-Miranda et al. [25], Hummel et al. [24], Alvelos et al. [22] and Aliti et al. [27].

Seven RCTs were included in the pooled analysis of heart failure hospitalization using a random-effects model comparing low versus high dietary sodium intake. The pooled effect showed 16% relative reduction in HF hospitalization (RR = 0.84; 95% CI: 0.62–1.15), but the results were not significant. Heterogeneity was low (*I*^2^ = 0%), indicating findings were consistent across studies. However, the confidence intervals were wide, indicating that a reduction in HF hospitalization ranged from clinically meaningful to no effect and statistical significance was not achieved. These findings suggest that neither strict, moderate, nor liberal sodium restriction demonstrated a statistically significant reduction in HF hospitalization in HF patients (Figure 4).

**Composite death/HF hospitalization**

**Figure 5:**
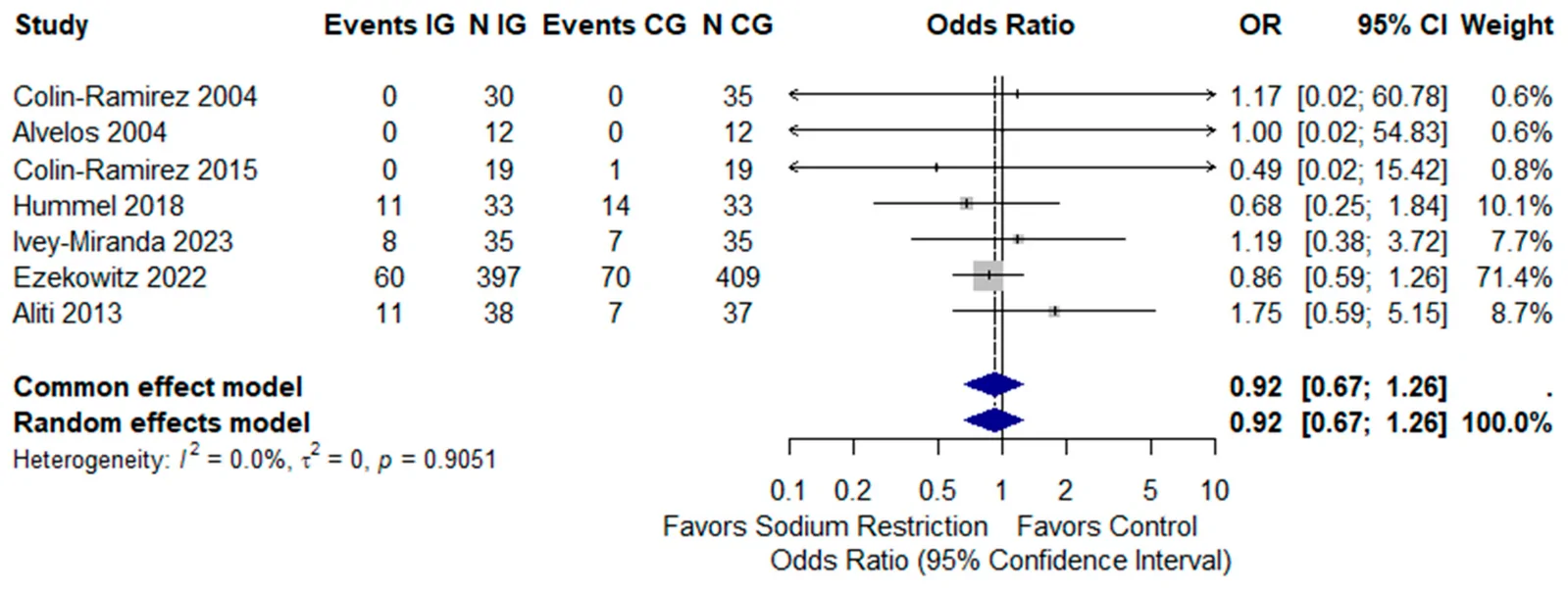
Composite of death and heart failure hospitalization. The trials shown include Alvelos et al. [22], Colin-Ramirez et al. [21], Colin-Ramirez et al. [23], Ezekowitz et al. [26], Ivey-Miranda et al. [25], Hummel et al. [24], Aliti et al. [27].

Seven RCTs were included in a pooled analysis of composite death/HF hospitalization using a random-effects model comparing lower versus high sodium intake. The pooled effect showed 8% lower odds of composite death/HF hospitalization with lower sodium intake (OR = 0.92; 95% CI: 0.67–1.26) compared to higher intake, but the results were not significant. The confidence intervals were wide, indicating that the impact could range from clinically meaningful to no effect. Heterogeneity was negligible (*I*^2^ = 0%); however, given the small number of contributing studies, this should not be interpreted as evidence of absence of clinically important heterogeneity. Overall, these findings suggest that neither strict, moderate, nor liberal sodium restriction significantly reduced composite death/HF hospitalization in HF patients (Figure 5).

Across outcomes, follow-up duration ranged from 5 days to 12 months. Since compatible time-to-event, person-time and common-time point data were inconsistently reported across trials, follow-up could not be fully standardized analytically. The pooled estimates should therefore be interpreted as effects observed across different study-specific follow-up periods rather than as estimates at a single prespecified time point.

**Quality of life (QoL)**

**Figure 6:**
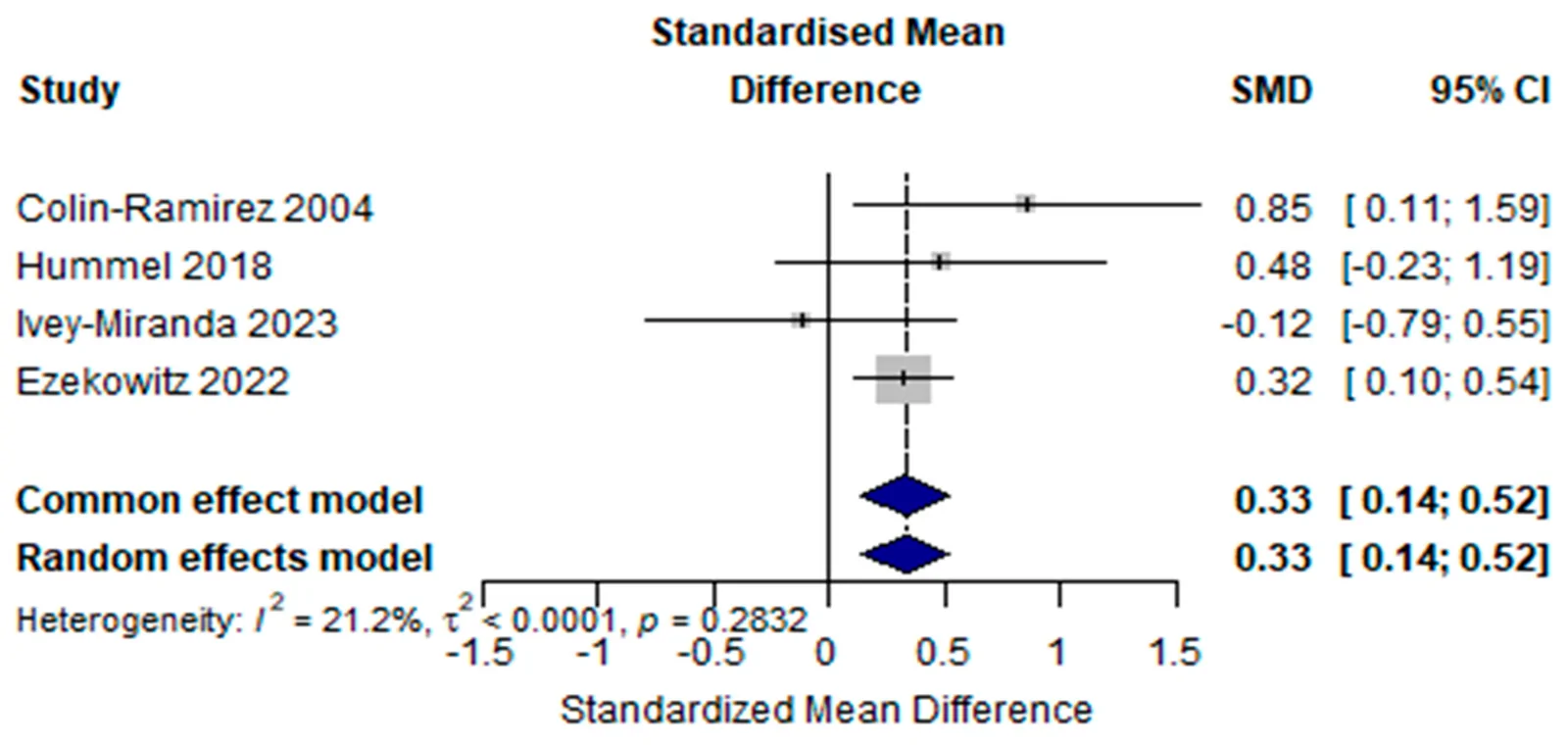
Quality of life. The trials shown include Colin-Ramirez et al. 2004 [21], Ezekowitz et al. [26], Ivey-Miranda et al. [25], and Hummel et al. [24]. additional trial labels shown in the figure are reported in Colin-Ramirez et al. [21].

Six RCTs were included in the pooled analysis of quality of life using a random-effects model comparing lower versus higher dietary sodium intake. Because different quality-of-life instruments were used across studies, effects were expressed as standardized mean differences (SMDs). Lower sodium intake was associated with improved quality of life (SMD = 0.33; 95% CI: 0.14–0.52), as the 95% confidence interval excluded the null value of 0. These estimates reflect QoL measurements obtained at the representative follow-up times reported by the included trials and may therefore combine short-term or long-term effects. Statistical heterogeneity was relatively low (*I*^2^ = 21.2%); however, these findings should not be interpreted as absence of clinical heterogeneity given differences among trials in sodium targets, populations, and follow-up durations (Figure 6).

**NYHA functional class**

**Figure 7:**
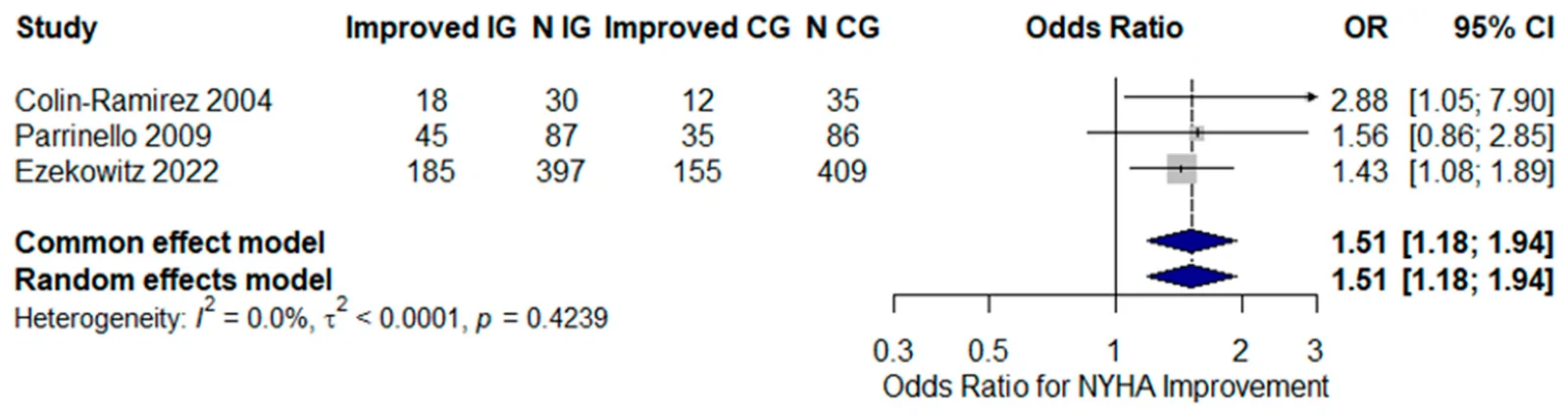
NYHA functional class. The trials shown include Colin-Ramirez et al. [21], Ezekowitz et al. [26] and Parrinello et al. [30]; the Colin-Ramirez and Parrinello trial labels shown in the figure are reported in Colin-Ramirez et al. [21].

Three RCTs were included in the pooled analysis of improvement in NYHA functional class using a common-effect Mantel-Haenszel odds-ratio model comparing lower versus higher sodium intake. Lower sodium intake was associated with higher odds of improvement in NYHA functional class (OR = 1.51; 95% CI: 1.18–1.94). The 95% confidence interval excluded the null value of 1.0, indicating a statistically significant association at the conventional 5% significance level. Because improvement was assessed at different study-specific endpoints, the effect estimate may reflect an effect across the reported follow-up periods rather than at a single time point. Statistical heterogeneity was low (*I*^2^ = 0%); however, with the small number of contributing trials, this estimate has limited power to detect between-study heterogeneity and should be interpreted cautiously (Figure 7).

**BNP/NT-proBNP**

**Figure 8:**
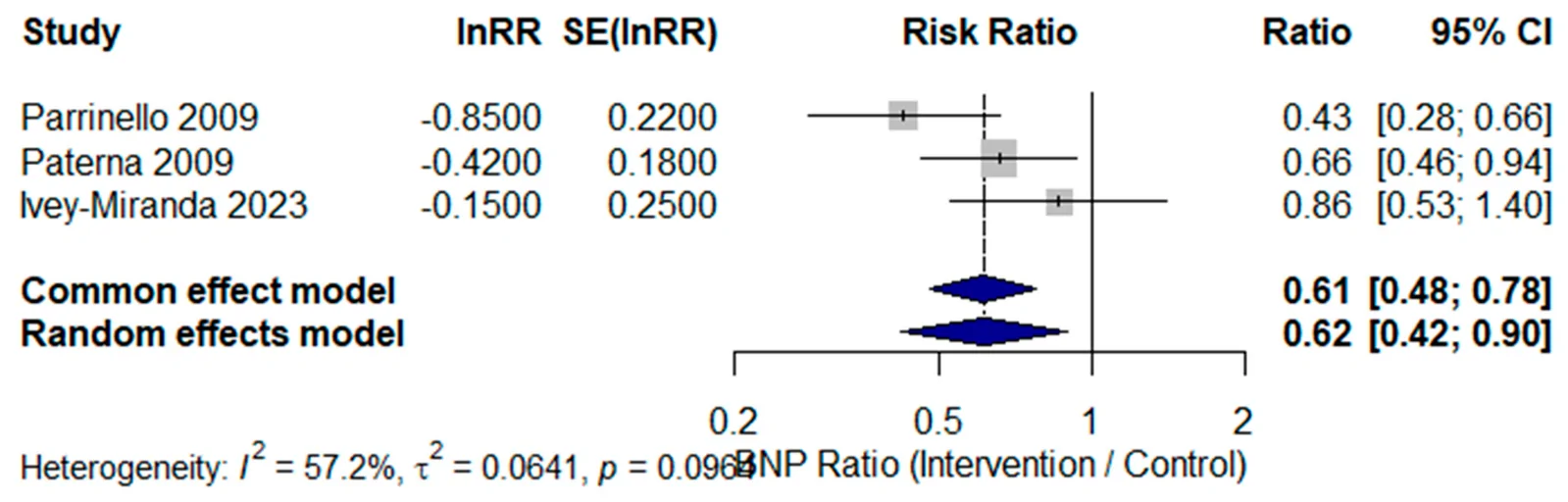
BNP/NT-proBNP. Forest plot of BNP/NT-proBNP. The trials shown include Parrinello et al. [30], Paterna et al. [29] and Ivey-Miranda et al. [25]; the Parrinello trial label shown in the figure is reported in Colin-Ramirez et al. [21].

Three RCTs were included in the pooled analysis of BNP/NT-proBNP ratio using a random-effects model comparing lower versus higher sodium intake. The pooled effect indicated that lower sodium intake was associated with a 38% relative reduction in B-type natriuretic peptide levels (ratio = 0.62; 95% CI: 0.42–0.90). The 95% CI corresponds to a 10% to 58% reduction in BNP/NT-proBNP levels with dietary sodium restrictions. The pooled estimate combines biomarker measurements obtained at different follow-up times; therefore, it may reflect both early and later biological responses to sodium restriction. Heterogeneity was moderate (*I*^2^ = 57.2%), indicating the variability across studies (Figure 8).

**Blood Pressure and Renal Function**

Pooled estimates of BP and renal function were not feasible because of insufficient data. None of the RCTs reported blood pressure. In regard to renal function, two RCTs by Parrinello et al. [30] reported worsening renal function or increased serum creatinine with moderate to liberal sodium intake (1800–2800 mg/day).

**Trial Sequential Analysis (TSA)**

**Figure 9:**
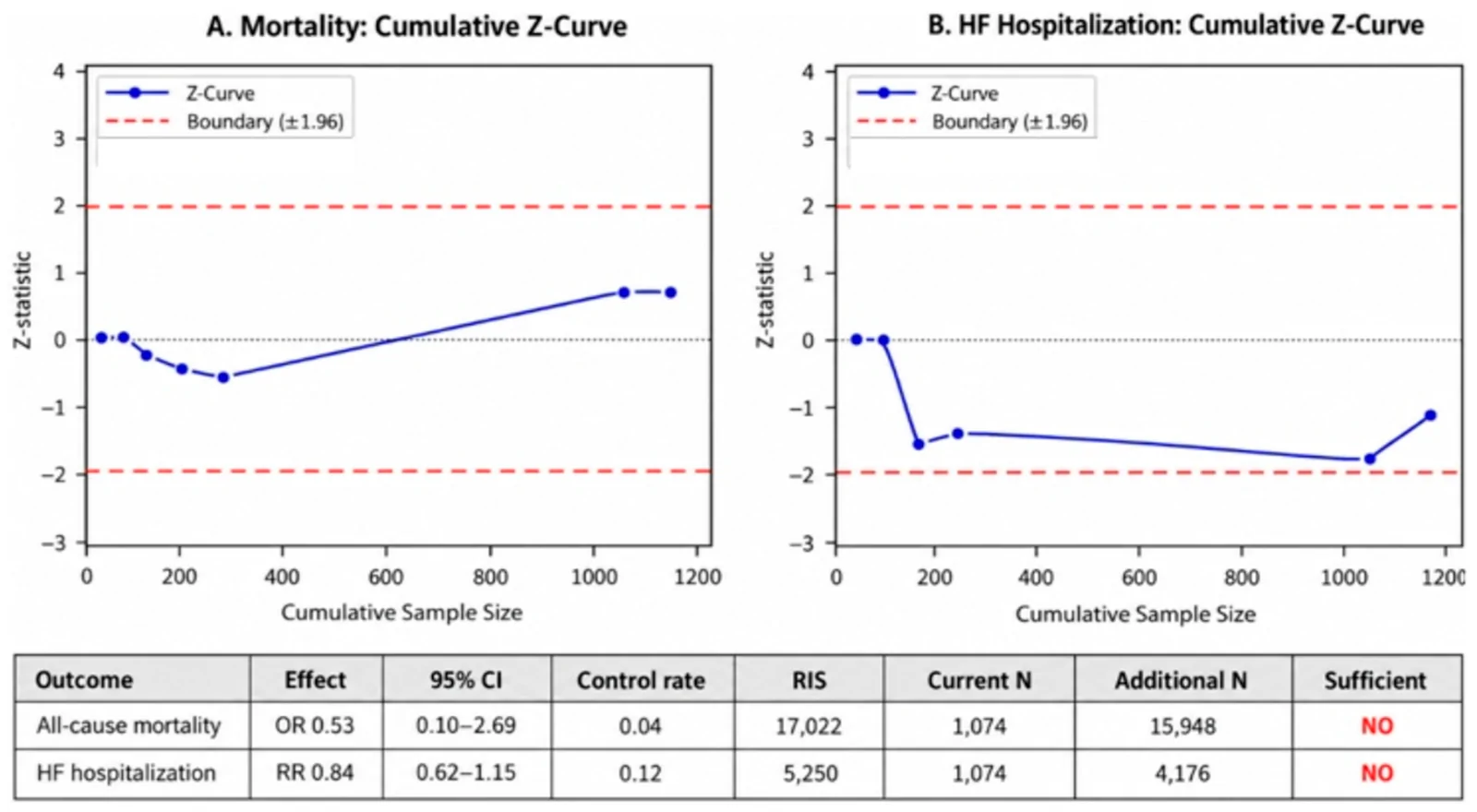
Cumulative Z-curves and required information size estimates for (**A**) all-cause mortality and (**B**) heart failure (HF) hospitalization. Blue lines represent the cumulative Z-curves, and horizontal red dashed lines indicate the conventional statistical significance thresholds (Z = ±1.96; two-sided α = 0.05). Effect estimates are OR 0.53 (95% CI 0.10–2.69) for all-cause mortality and RR 0.84 (95% CI 0.62–1.15) for HF hospitalization. Required information size (RIS) was estimated separately for each endpoint using a two-sided α = 0.05, β = 0.20 (80% power), an anticipated 20% relative risk reduction, equal allocation, and control event rates of 4% for mortality and 12% for HF hospitalization. The estimated RIS values were approximately 17,022 and 5250 participants, respectively. RIS lines are not displayed because these values exceed the horizontal-axis ranges; the values are provided in the accompanying table. With a current sample size of 1074 participants, approximately 15,948 and 4176 additional participants, respectively, would be needed to reach these information-size targets.

TSA for mortality and HF hospitalization showed that the current evidence was insufficient to draw definitive conclusions regarding the effect of different sodium intake targets on either endpoint. The cumulative Z-curves did not cross the conventional boundary for benefit (Z = ±1.96) or the futility boundary. Using endpoint-specific control event rates and the prespecified 20% RRR, the recalculated RIS was approximately 17,022 participants for all-cause mortality (control event rate 4%) and 5250 participants for HF hospitalization (control event rate 12%). The current information size was 1074 participants, leaving approximately 15,948 and 4176 additional participants required, respectively (Figure 9).

**Sub-Group Analyses**

***HFrEF-only versus mixed HFrEF/HFpEF populations***

**Figure 10:**
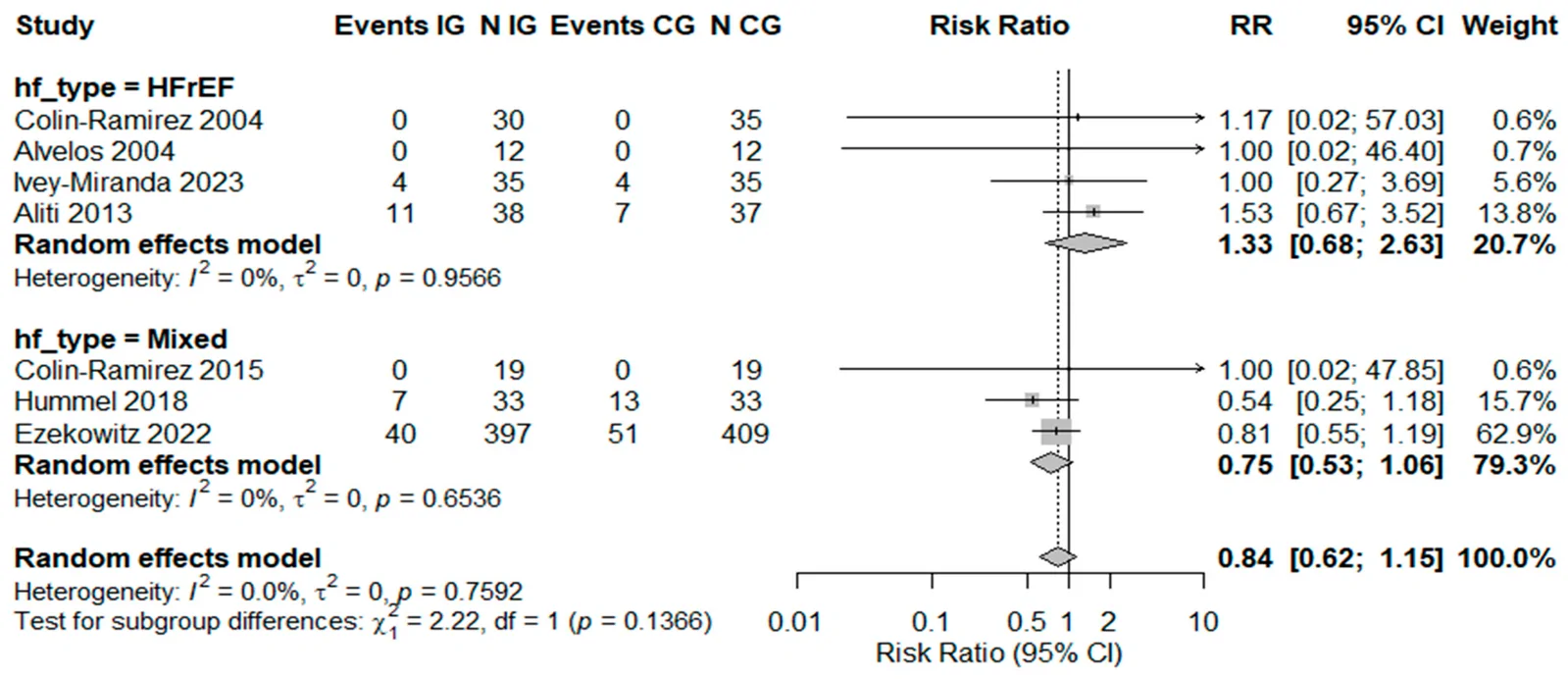
Subgroup analysis of HF hospitalization according to HF population: HFrEF-only versus mixed HFrEF/HFpEF trials. The trials shown include Colin-Ramirez et al. [21], Colin-Ramirez et al. [23], Ezekowitz et al. [26], Ivey-Miranda et al. [25], Hummel et al. [24], Alvelos et al. [22], and Aliti et al. [27]; additional trial labels shown in the figure are reported in Colin-Ramirez et al. [21].

Seven studies were included in the subgroup analysis by HF type for HF hospitalization using a random-effects model. There was no significant difference in HF between groups (X^2^ = 2.22; df = 1; *p* = 0.137). The pooled analysis of four RCTs that included HFrEF type patients showed a pooled risk ratio of RR = 1.33 (95% CI: 0.68–2.63), suggesting a non-significant association between sodium restriction and HF hospitalizations. The pooled analysis of three RCTs that included mixed (HFrEF/HFpEF) type patients showed a pooled risk ratio of RR = 0.77 (95% CI: 0.55–1.07), also suggesting a non-significant association between sodium restriction and HF hospitalizations. Heterogeneity was negligible (*I*^2^ = 0%); however, given the small number of contributing studies, this should not be interpreted as evidence of absence of clinically important heterogeneity. The wide confidence intervals suggested that the benefits of dietary sodium restrictions in HFrEF and mixed HF patients ranged from clinically meaningful to non-significant (Figure 10). This subgroup comparison contrasts trials that involved HFrEF-only patients with trials that included mixed HFrEF/HFpEF patients. Trials that involved pure HFpEF patients were not available.

**Sodium target**

**Figure 11:**
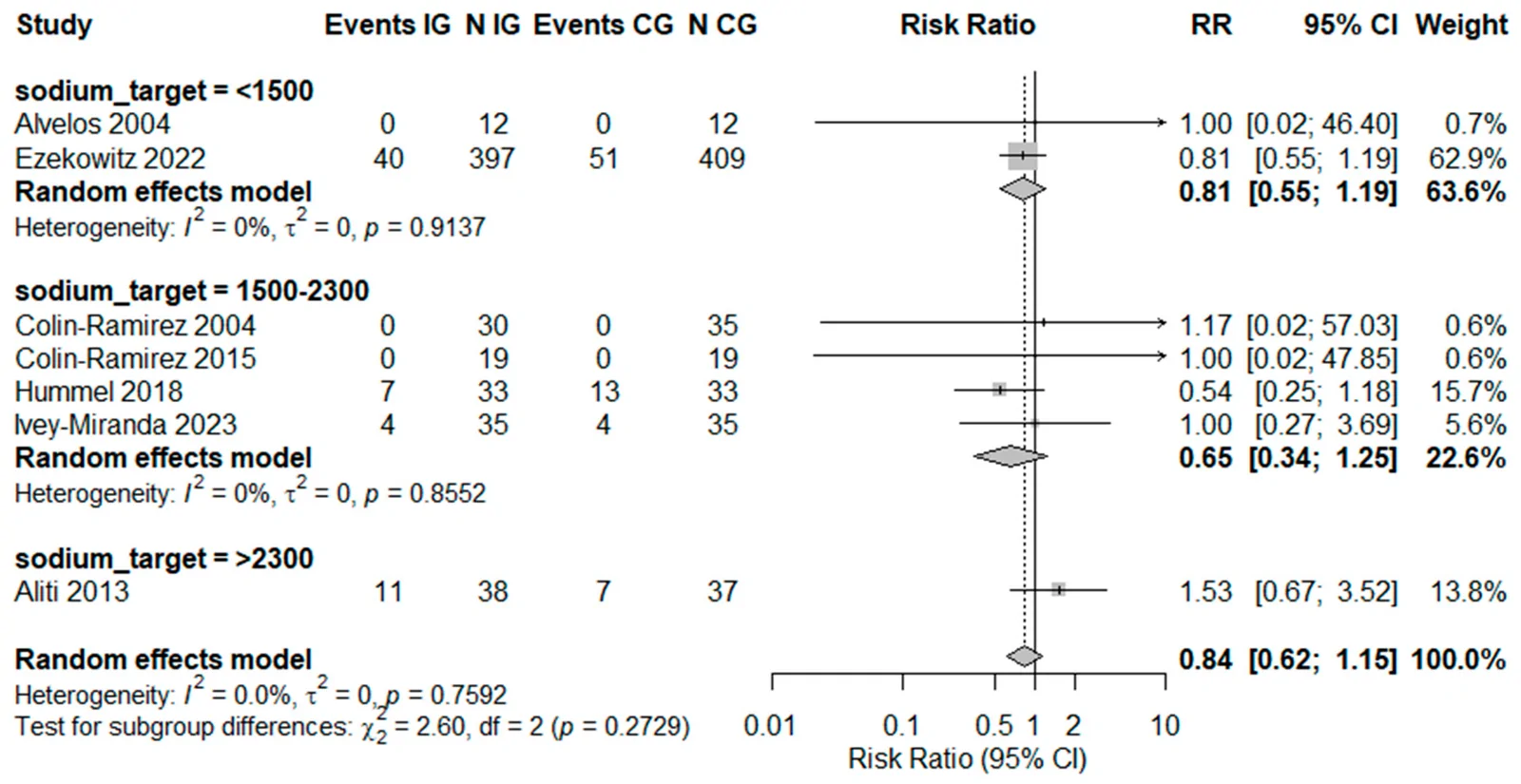
Sodium target (strict vs. moderate vs. liberal). The trials shown include Colin-Ramirez et al. [21], Colin-Ramirez et al. [23], Ezekowitz et al. [26], Ivey-Miranda et al. [25], Hummel et al. [24], Alvelos et al. [22], and Aliti et al. [27]; additional trial labels shown in the figure are reported in Colin-Ramirez et al. [21].

Seven studies were included in the subgroup analysis by sodium target for HF hospitalization using a random-effects model. Because most trials compared <2000 mg/day versus 2000–5000 mg/day sodium intake, the targets were mapped to our prespecified sodium categories based on the prescribed or achieved intake. There was no evidence of significant difference in effect between strict, moderate and liberal sodium intake groups (X^2^ = 2.60; df = 2; *p* = 0.273). The pooled analysis of two RCTs that used strict (<1500 mg/day) sodium intake showed a 19% relative reduction in HF hospitalization (RR = 0.81; 95% CI: 0.55–1.19). Pooled analysis of four RCTs that used moderate (1500–2300 mg/day) sodium intake showed a 35% relative reduction in HF hospitalization (RR = 0.65; 95% CI: 0.34–1.25). A single RCT that compared strict versus liberal (800 vs. 3000–5000 mg/day) showed a non-significant 53% increase in HF hospitalization with strict compared to liberal sodium intake (RR = 1.53; 95% CI: 0.67–3.52). Heterogeneity across groups was negligible (*I*^2^ = 0%, *p* = 0.273), indicating that there were no significant differences within subgroups (Figure 11).

**Meta-Regression Analysis**

**Figure 12:**
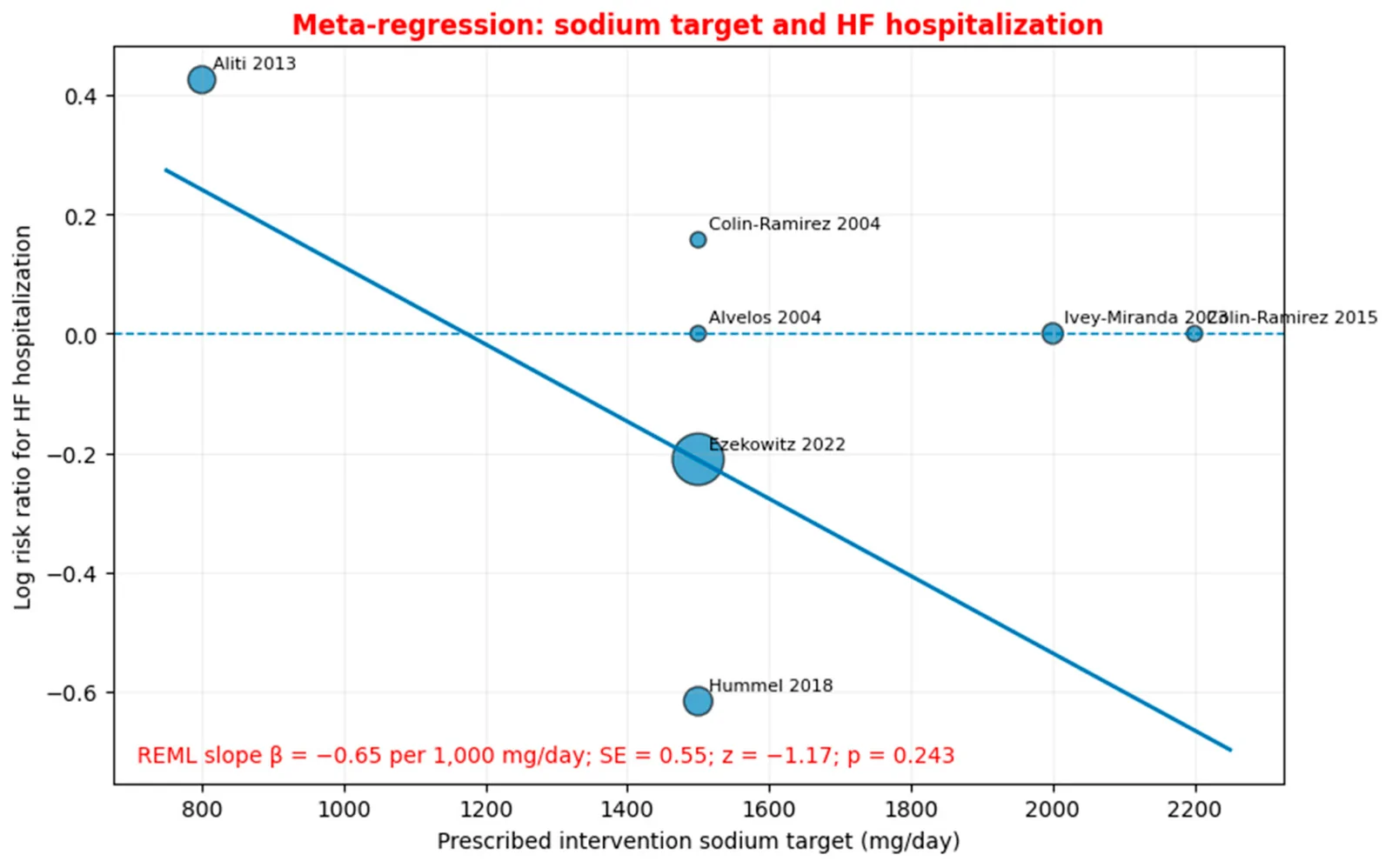
Random-effects meta-regression bubble plot for the association between prescribed sodium target and the effect on HF hospitalization. The x-axis shows the intervention sodium target (mg/day; midpoint used when a range was reported), and the y-axis shows the study log risk ratio. Bubble size is proportional to inverse-variance weight. REML meta-regression: β = −0.65 per 1000 mg/day (SE = 0.55), z = −1.17, *p* = 0.243. The trials shown include Colin-Ramirez 2004 [21], Colin-Ramirez 2015 [23], Ezekowitz et al. [26], Ivey-Miranda et al. [25], Hummel et al. [24], Alvelos et al. [22], Aliti et al. [27]; additional trial labels are reported in Colin-Ramirez et al. [21].

Meta-regression analysis was performed to explore whether the effect of sodium restriction on HF hospitalization varied according to the prescribed sodium target. For the continuous sodium-target analysis, the intervention target was expressed per 1000 mg/day; where a prescribed range was reported, its midpoint was used for this exploratory study-level analysis. Random-effects meta-regression using REML showed no statistically significant association between sodium target and the log risk ratio for HF hospitalization (slope β = −0.65 per 1000 mg/day; SE = 0.55; z = −1.17; *p* = 0.243). The corresponding bubble plot is presented in Figure 12, with bubble size proportional to inverse-variance weight. Given the small number of contributing studies and limited spread of sodium targets, this meta-regression is exploratory and should be interpreted cautiously.

**Sensitivity Analysis**

**Figure 13:**
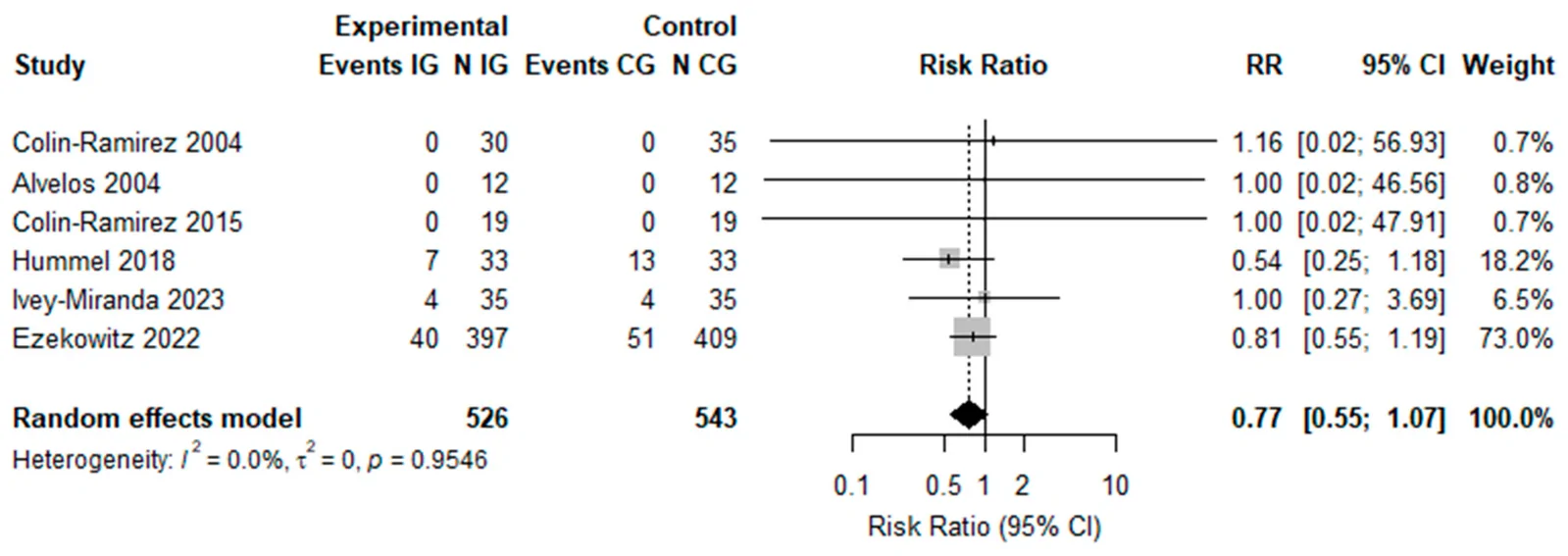
Sensitivity Analysis. The trials shown include Collin-Ramirez et al. [21], Collin-Ramirez et al. [23], Ezekowitz et al. [26], Ivey-Miranda et al. [25], Hummel et al. [24], and Alvelos et al. [22]; additional trial labels shown in the figure are reported in Colin-Ramirez et al. [21].

A sensitivity analysis excluding inpatient/acute HF studies showed a non-significant reduction in HF hospitalization among patients exposed to aggressive and moderate sodium intake compared to liberal intake (RR = 0.77; 95% CI: 0.55–1.07). Heterogeneity was negligible (*I*^2^ = 0%, *p* = 0.95) suggesting highly consistent findings across included outpatient and post-discharge studies. These findings show that excluding inpatient studies did not materially alter the direction or magnitude of the pooled effect estimate, indicating the robustness of primary findings (Figure 13).

**Leave-One-Out Analysis**

**Figure 14:**
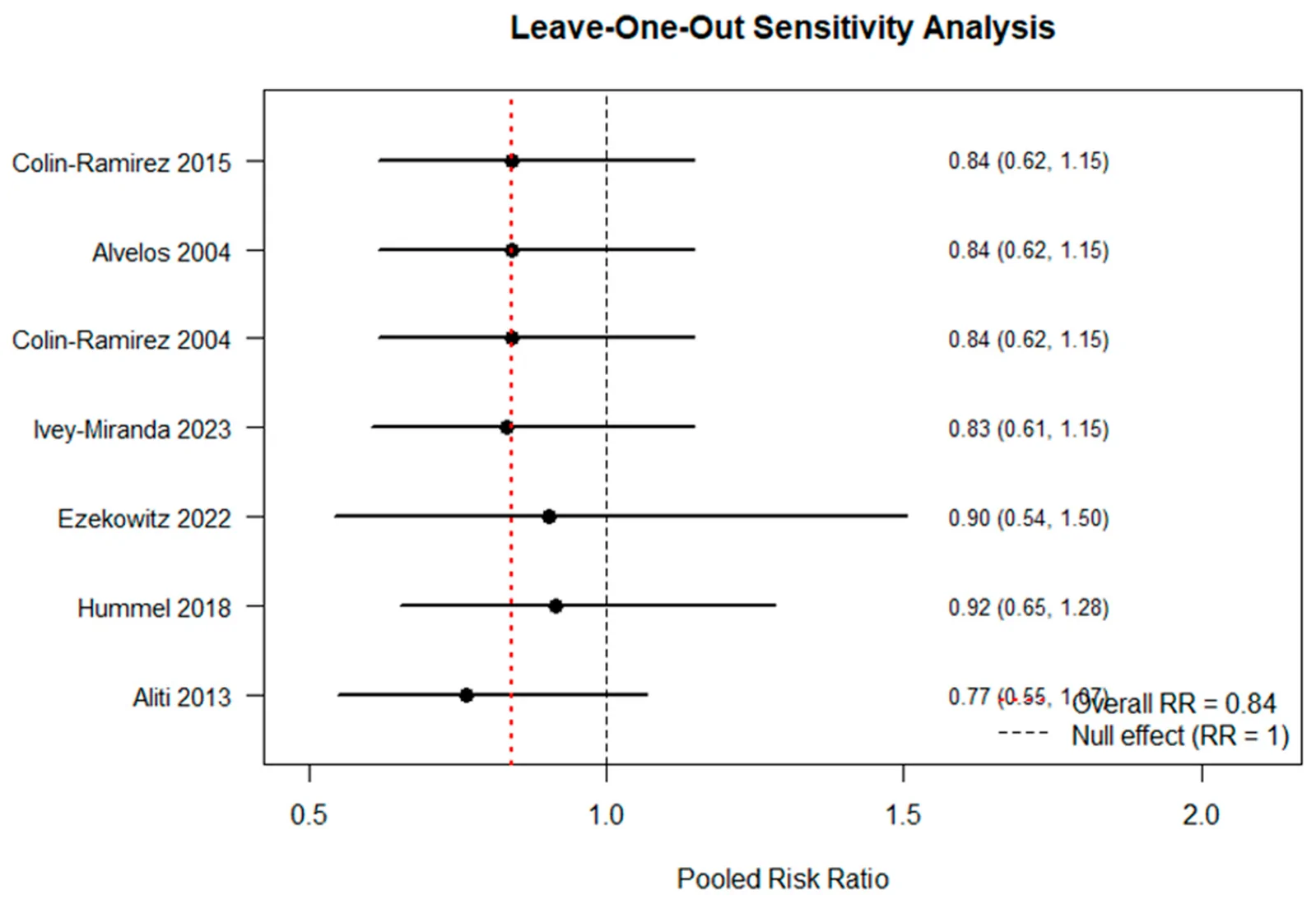
Leave-Out Analysis. The trials shown include Collin-Ramirez et al. [21], Collin-Ramirez et al. [23], Ezekowitz et al. [26], Ivey-Miranda et al. [25], Hummel et al. [24], Alvelos et al. [22], and Aliti et al. [27]; additional trial labels shown in the figure are reported in Colin-Ramirez et al. [21].

Leave-out analyses showed that the pooled RR for HF hospitalization remained stable ranging from 0.77 to 0.91 after removing each individual study compared with th primary pooled estimate of RR = 0.84 (95% CI: 0.62–1.15). Exclusion of the largest trial (SODIUM-HF) yielded a higher risk ratio RR = 0.90 while removing inpatient studies yielded a lower risk ratio of RR = 0.77. Confidence intervals of all pooled effect estimates crossed 1.0 suggesting the estimates were non-significant and no single study disproportionately influenced the overall result. These findings confirm the robustness of primary findings (Figure 14).

**Publication Bias**

**Figure 15:**
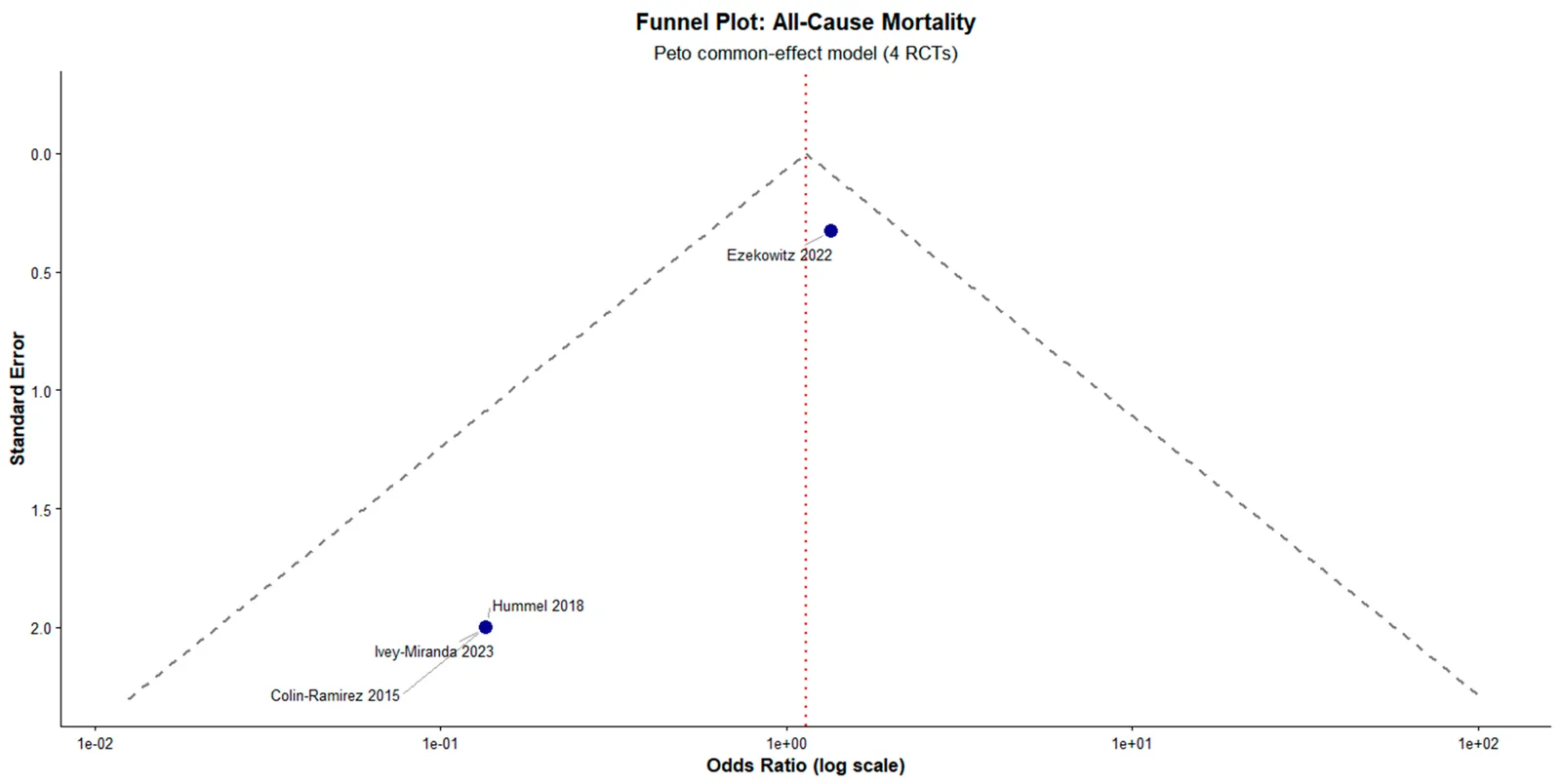
Publication Bias. The trials shown include Colin-Ramirez et al. Colin-Ramirez et al. [23], Ezekowitz et al. [26], Ivey-Miranda et al. [25], and Hummel et al. [24]; additional trial labels shown in the figure are reported in Colin-Ramirez et al. [21]. Scientific notation on the logarithmic x-axis is presented in ×10^n^ format.

Publication bias was assessed using a funnel plot for all-cause mortality based on 7 RCTs included in the meta-analysis. Because fewer than 10 studies contributed to the relevant meta-analysis, funnel-plot asymmetry and statistical tests for small-study effects have limited power and were considered exploratory. Accordingly, the absence of obvious funnel-plot asymmetry should not be interpreted as evidence that publication bias was absent. Most studies were clustered near the pooled odds ratio estimate though one large study [26] with a smaller standard error appeared closer to the funnel apex. Nevertheless, the small number (*n* = 7) of included studies limits the reliability of the funnel plot and of conducting Egger’s test (Figure 15).

## 4. Discussion

This meta-analysis examined the effects of strict versus moderate versus liberal dietary sodium intake in HF patients. The findings showed no statistically significant effect of sodium intake category on all-cause mortality, HF hospitalization, or composite outcome events in HF patients. However, strict to moderate sodium restrictions were associated with modest improvements in quality of life and NYHA functional class and lower BNP/NT-proBNP levels compared to liberal restrictions. Subgroup analyses by HF type and sodium target showed no significant trends toward a reduction in HF hospitalization. Heterogeneity was low for most outcomes. However, given the small number of studies contributing to each analysis, these estimates have limited power to detect between-study differences. A low *I*^2^ should not be interpreted as evidence of an absence of clinically relevant heterogeneity.

This review is consistent with the 2022 AHA/ACC/HFSA Heart Failure guidelines and 2023 ESC recommendations that continue to support dietary sodium restrictions in HF patients to improve metabolic health and reduce cardiovascular risks. The guidelines suggest that HF patients can benefit from moderate to individualized dietary sodium restrictions through the reduction in fluid retention, congestion symptoms and ventricular preload. Moreover, our findings are broadly consistent with this symptomatic focus but do not demonstrate a clear benefit on mortality or HF hospitalization. These mechanistic explanations are derived from prior guideline summaries and psychological studies rather than from the pooled randomized evidence in this review, which does not demonstrate clear benefits on mortality or HF hospitalization.

Large RCTs investigating dietary sodium restrictions in HF patients have failed to demonstrate a significant association between different dosages and all-cause mortality and readmission. The findings in this review showed no significant reduction in all-cause mortality with different dietary sodium intake targets in HF patients. Observational evidence has also examined associations between dietary sodium intake, mortality, and cardiovascular events in older adults [31]. A large trial (SODIUM_HF) by Ezekowitz et al. [26] reported no significant evidence that aggressive, modest or liberal sodium restrictions reduced death or hospitaliation. Paterna et al. [29] found that strict sodium intake (80 mmol/day) compared to usual care sodium intake (120 mmol/day) was associated with higher mortality, though the results were not significant. Neal et al. [32] found a linear association between average sodium intake and mortality. A long-term follow-up trial by Cook et al. [33] showed that strict to moderate sodium intake was associated with reduced mortality events and moderate sodium restriction may reduce extracellular fluid volume, systemic vascular resistance and BP, thereby lowering symptom burden and improving haemodynamic status. However, the improvements were not proportional to the degree of sodium restriction, which may explain the absence of benefits in hard clinical outcomes across studies. The more consistent signals of benefitwere observed for symptom-related and surrogate measures, including quality of life and NYHA functional class, for which the certainty of evidence was moderate at best.

Similarly, randomized trials have not demonstrated a correlation between dietary sodium restriction targets and HF hospitaliation. The findings in this review showed no significant reduction in HF hospitaliation with dietary sodium restrictions. Strict or moderate sodium restrictions did not demonstrate significant benefits in reducing HF admissions compared with liberal intakes. ubgroup analysis in this review showed no significant difference in hospitaliation across sodium targets or HF type. Individual RCTs have reported inconsistent results. Licata et al. [34] found that compared to moderate sodium restriction, strict restrictions (<1.8 g/day) increased hospitaliation and death. A review of smaller RCTs showed significant risks or harm depending on sodium doses. Aggressive sodium restriction was associated with a higher risk of composite outcome, but the effect on all-cause mortality or HF readmission was not significant [15].

Strict sodium restriction may offset intended benefits by triggering compensatory neurohormonal pathways, and activating the renin–angiotensin–aldosterone system (RAAS). This may result in vasoconstriction, impaired renal function and worsening haemodynamic stability. Paterna et al. [28] found that strict sodium restrictions were associated with an increase in the risk of adverse outcomes in HF patients including worse cardiac stress, impaired renal function and increased HF hospitalization.

In this review, findings showed that strict to moderate sodium restrictions had modest benefits in improving QoL and NYHA functional class. Colin-Ramirez et al. [21] found improvement in QoL and NYHA functional class with moderate sodium restrictions of 2000–3000 mg/day compared to <2000 mg/day. According to Ezekowitz et al. [26], different sodium doses did not reduce readmissions or mortality but improved QoL and NYHA functional class. Patel et al. [35] noted that HF patients may experience symptomatic benefits and functional improvement even without reductions in mortality or hospitaliation risks.

While the data were insufficient to examine the metabolic health of HF patients across sodium intake targets, two studies reported worsening renal function. Paterna et al. [28] found that lower sodium intake increases serum creatinine, while Ivey-Miranda et al. [25] found that different sodium doses did not influence metabolic health. Although evidence regarding metabolic health status is limited, the included studies indicated that both liberal and strict sodium intakes may have been associated with impaired renal function and haemodynamic regulation in selected HF patients. On the other hand, strict sodium intake may alter fluid load and influence sodium function impairment. Taken together, these findings suggest that moderate sodium restriction may offer symptomatic benefits for some patients with HF, but current evidence is insufficient to define an optimal sodium target or demonstrate reductions in mortality and HF hospitalization.

### Limitations

Several limitations should be considered. First, the number of trials contributing to individual outcomes was small, and several trials had small sample sizes or few/zero events, resulting in imprecise estimates for major clinical outcomes. Second, the included trials differed in prescribed sodium targets, HF phenotypes, clinical settings, concomitant interventions, and follow-up durations, introducing important clinical heterogeneity despite low statistical heterogeneity for several outcomes. Third, one trial included patients with acute decompensated HF, whereas most studies evaluated stable or chronic HF populations; although a sensitivity analysis excluding acute/inpatient HF did not materially change the direction of the hospitalization estimate, this difference limits direct comparability. Fourth, the HF phenotype subgroup analysis compared HFrEF-only trials with mixed HFrEF/HFpEF populations and therefore cannot establish differential treatment effects between HFrEF and HFpEF. Fifth, meta-regression and assessment of small-study effects were based on a limited number of trials and should be considered exploratory. Sixth, differences in outcome definitions and follow-up durations may have affected pooled estimates. Finally, data regarding renal function, blood pressure, adverse events, and other safety outcomes were insufficient for robust quantitative synthesis. These limitations reduce the certainty with which an optimal sodium target can be identified.

Follow-up duration varied substantially across trials, ranging from 5 days to 12 months. Individual participant-time data were unavailable, precluding the calculation of standardized incidence rates or time-to-event effect estimates across studies. While pooling was done with sufficiently comparable clinical outcome definitions, unequal follow-up could not be fully addressed analytically. Pooling event-based outcomes across unequal observation periods may influence estimates of mortality, HF hospitalization and the composite outcome because endpoint RRs reflect cumulative risk over the study-specific observation periods. It may have combined short-term and long-term effects for QoL, NYHA class and BNP/NT-proBNP. Therefore, the pooled estimates of these outcomes should be interpreted as trial-level effects over heterogeneous follow-up periods rather than effects at a uniform time point.

## 5. Conclusions

In this systematic review and meta-analysis of randomized controlled trials, lower dietary sodium targets did not significantly reduce all-cause mortality, HF hospitalization, or the composite of death and HF hospitalization compared with higher sodium intake. However, lower sodium intake was associated with improvements in symptom-related and functional outcomes, including quality of life and NYHA functional class, as well as lower BNP/NT-proBNP levels. These findings should be interpreted cautiously because of differences in sodium targets, HF populations, clinical settings, follow-up durations, and the limited number of trials contributing to several analyses. The available evidence does not establish that strict or moderate sodium restriction is superior for major clinical outcomes and is insufficient to define a single optimal sodium target for all patients with HF. Further adequately powered RCTs using standardized sodium targets and clinically relevant long-term outcomes are needed.

## Figures and Tables

**Table 1:** PRISMA checklist.

Section and Topic	Item #	Checklist Item	Location Where Item Is Reported
**Title**			
Title	1	Identify the report as a systematic review.	p. 1
**Abstract**			
Abstract	2	See the PRISMA 2020 for Abstracts checklist.	p. 1
**Introduction**			
Rationale	3	Describe the rationale for the review in the context of existing knowledge.	p. 2
Objectives	4	Provide an explicit statement of the objective(s) or question(s) the review addresses.	p. 2
**Methods**			
Eligibility criteria	5	Specify the inclusion and exclusion criteria for the review and how studies were grouped for the syntheses.	p. 3
Information sources	6	Specify all databases, registers, websites, organizations, reference lists and other sources searched or consulted to identify studies. Specify the date when each source was last searched or consulted.	p. 3
Search strategy	7	Present the full search strategies for all databases, registers and websites, including any filters and limits used.	p. 3
Selection process	8	Specify the methods used to decide whether a study met the inclusion criteria of the review, including how many reviewers screened each record and each report retrieved, whether they worked independently, and if applicable, details of automation tools used in the process.	p. 3
Data collection process	9	Specify the methods used to collect data from reports, including how many reviewers collected data from each report, whether they worked independently, any processes for obtaining or confirming data from study investigators, and if applicable, details of automation tools used in the process.	p. 3
Data items	10a	List and define all outcomes for which data were sought. Specify whether all results that were compatible with each outcome domain in each study were sought (e.g., for all measures, time points, analyses), and if not, the methods used to decide which results to collect.	p. 3
	10b	List and define all other variables for which data were sought (e.g., participant and intervention characteristics, funding sources). Describe any assumptions made about any missing or unclear information.	p. 3
Study risk of bias assessment	11	Specify the methods used to assess risk of bias in the included studies, including details of the tool(s) used, how many reviewers assessed each study and whether they worked independently, and if applicable, details of automation tools used in the process.	p. 3
Effect measures	12	Specify for each outcome the effect measure(s) (e.g., risk ratio, mean difference) used in the synthesis or presentation of results.	p. 4
Synthesis methods	13a	Describe the processes used to decide which studies were eligible for each synthesis (e.g., tabulating the study intervention characteristics and comparing against the planned groups for each synthesis (item #5)).	p. 4
	13b	Describe any methods required to prepare the data for presentation or synthesis, such as handling of missing summary statistics, or data conversions.	p. 4
	13c	Describe any methods used to tabulate or visually display results of individual studies and syntheses.	p. 4
	13d	Describe any methods used to synthesize results and provide a rationale for the choice(s). If meta-analysis was performed, describe the model(s), method(s) to identify the presence and extent of statistical heterogeneity, and software package(s) used.	p. 4
	13e	Describe any methods used to explore possible causes of heterogeneity among study results (e.g., subgroup analysis, meta-regression).	p. 4
	13f	Describe any sensitivity analyses conducted to assess robustness of the synthesized results.	p. 4
Reporting bias assessment	14	Describe any methods used to assess risk of bias due to missing results in a synthesis (arising from reporting biases).	p. 4
Certainty assessment	15	Describe any methods used to assess certainty (or confidence) in the body of evidence for an outcome.	p. 4
**Results**			
Study selection	16a	Describe the results of the search and selection process, from the number of records identified in the search to the number of studies included in the review, ideally using a flow diagram.	p. 4
	16b	Cite studies that might appear to meet the inclusion criteria, but which were excluded, and explain why they were excluded.	
Study characteristics	17	Cite each included study and present its characteristics.	p. 5
Risk of bias in studies	18	Present assessments of risk of bias for each included study.	pp. 6–7
Results of individual studies	19	For all outcomes, present, for each study: (a) summary statistics for each group (where appropriate) and (b) an effect estimate and its precision (e.g., confidence/credible interval), ideally using structured tables or plots.	pp. 7–13
Results of syntheses	20a	For each synthesis, briefly summarize the characteristics and risk of bias among contributing studies.	P.7–13
	20b	Present results of all statistical syntheses conducted. If meta-analysis was done, present for each the summary estimate and its precision (e.g., confidence/credible interval) and measures of statistical heterogeneity. If comparing groups, describe the direction of the effect.	pp. 7–13
	20c	Present results of all investigations of possible causes of heterogeneity among study results.	pp. 13–14
	20d	Present results of all sensitivity analyses conducted to assess the robustness of the synthesized results.	pp. 13–14
Reporting biases	21	Present assessments of risk of bias due to missing results (arising from reporting biases) for each synthesis assessed.	NA
Certainty of evidence	22	Present assessments of certainty (or confidence) in the body of evidence for each outcome assessed.	
**Discussion**			
Discussion	23a	Provide a general interpretation of the results in the context of other evidence.	pp. 15–16
	23b	Discuss any limitations of the evidence included in the review.	p. 16
	23c	Discuss any limitations of the review processes used.	p. 16
	23d	Discuss implications of the results for practice, policy, and future research.	p. 16
**Other Information**			
Registration and protocol	24a	Provide registration information for the review, including register name and registration number, or state that the review was not registered.	
	24b	Indicate where the review protocol can be accessed, or state that a protocol was not prepared.	
	24c	Describe and explain any amendments to information provided at registration or in the protocol.	
Support	25	Describe sources of financial or non-financial support for the review, and the role of the funders or sponsors in the review.	
Competing interests	26	Declare any competing interests of review authors.	
Availability of data, code and other materials	27	Report which of the following are publicly available and where they can be found: template data collection forms; data extracted from included studies; data used for all analyses; analytic code; any other materials used in the review.	

From: Page MJ, McKenzie JE, Bossuyt PM, Boutron I, Hoffmann TC, Mulrow CD et al. The PRISMA 2020 statement: An updated guideline for reporting systematic reviews. BMJ 2021;372:n71. doi: 10.1136/bmj.n71. This work is licensed under CC BY 4.0. To view a copy of this license, visit https://creativecommons.org/licenses/by/4.0/.

**Table 2:** Characteristics of included studies.

Study	Design	HF Type	Setting	Sodium Level (mg/day)	N	Age (Mean)	Sex (% Male)	Follow-up
Colin-Ramirez 2004 [21]	Pilot RCT	HFrEF	Outpatient	1500/2300	29/29	62.1	36%	6 mo
Alvelos 2004 [22]	Parallel RCT	HFrEF	Outpatient	<1500/2300	12//12	69.9	71%	15 d
Colin-Ramirez 2015 [23]	Parallel RCT	HFrEF + HFpEF	Outpatient	2000–2400/>2400 (usual)	86/117	65.5/66.3	70%/74%	12 mo
Hummel 2018 [24] (GOURMET-HF)	Single-blind RCT	HFrEF + HFpEF	Outpatient (post-discharge)	1500 (DASH)/usual care	33/33	71	70%	12 wk
Ivey-Miranda 2023 [25]	Double-blind RCT	HFrEF	Outpatient	2000/3000	35/35	61/58	54%/82%	20 wk
Ezekowitz 2022 [26]	Open-label RCT	HFrEF + HFpEF	Outpatient	<1500/usual care	397/409	67	66%	12 mo
Aliti 2013 [27]	Parallel RCT	HFrEF	Inpatient/ADHF	800/3000–5000	38/37	NR	NR	30 d
Paterna 2008 [28]	RCT	HFrEF	Compensated	1800 (80 mmol)/2800 (120 mmol)	114/118	55–83	62%	6 mo
Paterna 2009 [29]	RCT	HFrEF	Compensated	1800 (80 mmol)/2800 (120 mmol)	87/86	74–77	37%	12 mo
Parrinello 2009 [30]	RCT	HFrEF	Compensated	1800/2800	87/86	72	61%	12 mo

**Table 3:** GRADE Summary of findings.

Outcome	No. of Studies	Effect Estimate (95% CI)	Control Event Rate	Absolute Effects (95% CI)	Certainty of Evidence (GRADE)	Reasons for Downgrading
All-cause mortality	7 RCTs	OR 0.53 (0.10 to 2.69)	35 per 1000	16 fewer per 1000	Low	Downgraded one level for risk of bias (some studies with high or some concerns in RoB 2 domains); downgraded one level for imprecision (very wide CI, few events, CI includes both substantial benefit and harm).
HF hospitalization	7 RCTs	RR 0.84 (0.62 to 1.15)	45 per 1000	7 fewer per 1000	Low	Downgraded one level for risk of bias (outcome-level RoB 2 assessments with some concerns/high risk in several trials); downgraded one level for imprecision (CI includes no effect and a clinically meaningful reduction).
Composite death/HF hospitalization	7 RCTs	OR 0.92 (0.67 to 1.26)	171 per 1000	11 fewer per 1000	Low	Downgraded one level for risk of bias; downgraded one level for imprecision (CI includes no effect and a potentially important reduction).
Quality of life (QoL)	6 RCTs	SMD 0.33 (0.15 to 0.51)	N/A	Continuous outcome	Moderate	Downgraded one level for risk of bias (measurement issues, attrition, and use of different QoL instruments across trials); not downgraded for imprecision as the CI excludes no effect.
NYHA functional class (improvement)	3 RCTs	OR 1.51 (1.18 to 1.94)	381 per 1000	101 more per 1000	Moderate	Downgraded one level for risk of bias (few studies, some concerns in RoB 2 domains); not downgraded for imprecision as the CI excludes no effect.

## Data Availability

The analyzed dataset in this research study can be provided by the corresponding author upon reasonable request.
